# Supplementary material for: The importance of spatial language for early numerical development in preschool: Going beyond verbal number skills
Source: PLoS One. 2023 Sep 29;18(9):e0292291. doi: 10.1371/journal.pone.0292291 (PMC10540965; doi:10.1371/journal.pone.0292291)
Supplement: Attachment — Submitted filename: Review of PONE-D-23-11288R1.docx [file pone.0292291.s002.docx]

Review of: PONE-D-23-11288R1

Given the length of this manuscript (the download was 155 pages) and the short time provided to review it (10 days), I am going to presume that the expectation was to see if the queries of prior reviewers were met. If this was the purpose of an additional review, then indeed, questions brought up by prior reviewers seem to be met by the author.

In this reviewer’s opinion however, the manuscript seems excessively long and arduous to read. Several concepts that appear to be tangentially related (e.g., phonological awareness, spatial awareness, number mapping, language exposure) that would likely come together in the course of normal child development are studied for their relation to one another making the manuscript appear to lack true direction and purpose. The reference to deaf children and the statement that deaf learners may not truly be visual learners (page 49) seems out of place and not grounded in any finding that would emerge from the research in this study. This study did not include deaf children so statements of this nature cannot be made.

The manuscript would benefit from being drastically cut and streamlined- if the topic of focus is the relationship between children’s use of spatial language and how this related to their early numerical skills, then focus on this and only this.
