# Supplementary material for: The importance of spatial language for early numerical development in preschool: Going beyond verbal number skills
Source: PLoS One. 2023 Sep 29;18(9):e0292291. doi: 10.1371/journal.pone.0292291 (PMC10540965; doi:10.1371/journal.pone.0292291)
Supplement: Attachment — Submitted filename: spatial language and math .docx [file pone.0292291.s003.docx]

The reviewer and I both agree you should take this section out. There is work that you are not familiar with that make parts of this problematic.

For example Deaf children who SIGN have better spatial skills so if someone know this and that phonological awareness is not really used by deaf readers it might be interesting to see the differences.

Maybe one sentence or two in future research should do it and make it ready to go.

Finally, it is important to remind that the present findings might only apply to healthy

young children without learning disabilities (e.g., dyscalculia) and/or sensory impairments

(e.g., deafness). Whether or not the current pattern of results stressing the crucial role of

linguistic factors like spatial language and phonological awareness in early numerical

development in preschool can be generalized to young children not using a conventional

spoken language due to very little or no functional hearing should be assessed in future studies.

Notably, previous studies have consistently shown that deaf and hard-of-hearing

children, not exposed to fluent sign language from birth, generally show severe and persistent

difficulties in numerical, arithmetical and mathematical skills compared to their hearing peers

from preschool onward [148-154]. Especially, tasks requiring symbolic as opposed to

nonsymbolic quantity processing seem more challenging (e.g., [155]). Their delay also appears

most pronounced on verbal numerical tasks, including counting [156], fractions [157], problem

solving [158], and arithmetic skills, notably multiplications (e.g., [159]), compared to

visuospatial tasks, such as geometry (e.g., [158]). In line with the current findings, the

difficulties of deaf pupils were assumed to be due amongst others to their lower language

abilities early in development and/or their lack of exposure to spontaneous numerical language

in their home environment (for a review, see [160]). Importantly, despite their language

impairment, deaf individuals were previously reported to show distinct advantages in various

visual domains, including visual attentional shifting and scanning [161], peripheral detection

of motion (e.g., [162]), as well as the manipulation of mental images (e.g., [163]). They were

SPATIAL LANGUAGE AND EARLY NUMERICAL SKILLS

49

also reported to benefit from instructions emphasizing spatial representations and strategies

when solving numerical tasks. Namely, Zarfaty et al. [164] showed that 3- to 4-year-old deaf

children outperformed their hearing peers on a task requiring the recollection of the number of

items in a set when presented simultaneously in a spatial array but not when displayed

sequentially in a temporal sequence. Similarly, Chen and Wang [165] showed that general

cognitive abilities such as spatial abilities and processing speed were better predictors of

mathematical performances in deaf or hard-of-hearing students than nonsymbolic and symbolic

numerical magnitude processing, thereby likely implying the particular importance of spatial

strategies for mathematical achievement in those individuals. Promoting the use of such

strategies in deaf children might thus be more beneficial for early numerical development and

yield greater gains than stressing the use of spatial language and/or phonological awareness in

that population.

Nonetheless, the relative contributions of spatial language, phonological awareness and

spatial abilities to early numerical development in deaf children needs further investigation.

More concretely, it should be assessed whether or not deaf or hard-of-hearing individuals are

in fact true visual learners (but see, [166]) in that most of the early numerical abilities included

in the present study are actually predicted by spatial abilities rather than phonological

awareness and/or spatial language. One should also determine if potential performance

differences of deaf pupils with respect to their hearing peers could then be explained by their

preferential use of visuospatial strategies on numerical tasks primarily predicted by verbal

factors, such as spatial language, in healthy children. Notably, Buyle and colleagues [159]

recently speculated that the lower performances of deaf individuals particularly on

multiplication but not subtraction problems might be due to their reliance on a visuospatial as

opposed to verbal route when solving multiplications. This visuospatial detour might impose a

greater cognitive load and thereby explain slower performances in deaf individuals.

SPATIAL LANGUAGE AND EARLY NUMERICAL SKILLS
