# Supplementary material for: The importance of spatial language for early numerical development in preschool: Going beyond verbal number skills
Source: PLoS One. 2023 Sep 29;18(9):e0292291. doi: 10.1371/journal.pone.0292291 (PMC10540965; doi:10.1371/journal.pone.0292291)
Supplement: Attachment — Submitted filename: Response to Reviewers.docx [file pone.0292291.s004.docx]

Dear Editor, Prof. Dr Mary Diane Clark,

Dear Reviewer,

Thank you for the positive evaluation of the revision of our manuscript. Our responses are in purple.

**Additional Editor Comments:**

Please see the section that the reviewer and I would like to see deleted. Other changes were fine.

I am attaching what the reviewer and I think you should take out of this version. Sorry my suggestion got you into a rabbit hole. There are other issues and I would love to see this done with young deaf children by a researcher who is aware of the current publications but some of your statements disagree with these new publications.

So, if you take out what is in the attachment, we should be ready to go.

Thank you for this feedback. We have now deleted the paragraph about deaf individuals, as indicated in the attachment. As suggested by the Editor, we have now only briefly introduced the topic of deafness in children by concluding the “Limitations and future directions” section with the following:

“Finally, it is important to remind that the present findings might only apply to typical young children without learning disabilities (e.g., dyscalculia) and/or sensory impairments (e.g., deafness). Future studies should extend the present findings by determining whether the current pattern of results also holds in atypical populations, such as for instance deaf children, who differ in their spatial abilities and reliance on phonological awareness skills”.

If the Editor would prefer us to remove this short paragraph as well, please let us know.

**Reviewer Comments:**

Given the length of this manuscript (the download was 155 pages) and the short time provided to review it (10 days), I am going to presume that the expectation was to see if the queries of prior reviewers were met. If this was the purpose of an additional review, then indeed, questions brought up by prior reviewers seem to be met by the author.

Thank you for this positive feedback.

In this reviewer’s opinion however, the manuscript seems excessively long and arduous to read. Several concepts that appear to be tangentially related (e.g., phonological awareness, spatial awareness, number mapping, language exposure) that would likely come together in the course of normal child development are studied for their relation to one another making the manuscript appear to lack true direction and purpose. The reference to deaf children and the statement that deaf learners may not truly be visual learners (page 49) seems out of place and not grounded in any finding that would emerge from the research in this study. This study did not include deaf children so statements of this nature cannot be made.

The manuscript would benefit from being drastically cut and streamlined - if the topic of focus is the relationship between children’s use of spatial language and how this related to their early numerical skills, then focus on this and only this.

As also suggested by the Editor, we have now removed the paragraph about deaf individuals in the “Limitations and future directions” section to make this manuscript more focussed on the importance of spatial language for early numerical skills in typical child development.
